# Fracture Strength and Marginal Adaptation of Conservative and Extended MOD Cavities Restored with Cention N

**DOI:** 10.1155/2021/5599042

**Published:** 2021-07-06

**Authors:** Maryam Firouzmandi, Ali Asghar Alavi, Dana Jafarpour, Soroush Sadatsharifee

**Affiliations:** ^1^Oral and Dental Disease Research Center, Department of Operative Dentistry, School of Dentistry, Shiraz University of Medical Sciences, Shiraz, Iran; ^2^Department of Operative Dentistry, Biomaterials Research Center, School of Dentistry, Shiraz University of Medical Sciences, Shiraz, Iran; ^3^Biomaterials Research Center, School of Dentistry, Shiraz University of Medical Sciences, Shiraz, Iran; ^4^Undergraduate Student, Department of Operative Dentistry, School of Dentistry, Shiraz University of Medical Sciences, Shiraz, Iran

## Abstract

The aim of the present study was to compare the fracture strength and marginal adaptation of MOD cavities restored with Cention N, bonded Cention N, and resin composite, as well as to investigate the effect of cavity preparation volume on those properties. In this experimental study, 120 human maxillary premolars were randomly divided into six groups according to the type of restoration and cavity volume (*n* = 20): (I) conservative MOD restored with Cention N, (II) conservative MOD restored with bonded Cention N, (III) conservative MOD restored with Z250 resin composite, (IV) extended MOD restored with Cention N, (V) extended MOD restored with bonded Cention N, and (VI) extended MOD restored with Z250 resin composite. Fracture strength (MPa) was tested using a universal testing machine. To investigate marginal adaptation, polyvinyl-siloxane impressions were taken and poured with epoxy resin. Resin replicas were examined by SEM (×400) for marginal adaptation. ANOVA tests, Tukey's test, and independent *t*-test were used to analyze data (*P* ≤ 0.05). Among conservative restorations, the fracture strength of bonded Cention N was significantly greater than that of Cention N (*P* = 0.001), while in the extended preparations, there was no significant difference between fracture strengths of different types of restorations (*P* = 0.579). In terms of marginal adaptation, there was no significant difference between different types of conservative restorations (*P* = 0.232). However, in extended preparations, composite showed significantly lower marginal adaptation than Cention N and bonded Cention N (*P* = 0.004 and *P* = 0.045, respectively). Conservative preparations showed significantly greater fracture strength and marginal adaptation compared to extended ones in groups restored with composite. The volume of cavity preparation was shown to be effective in the materials fracture strength and marginal adaptation. Cention N showed promising results in terms of fracture strength and marginal adaptation.

## 1. Introduction

Increasing attention to esthetic dentistry has led to the widespread use of composite resins, not only as a direct restorative material in anterior teeth but also as a potential material of choice to substitute for unaesthetic amalgam restorations in posterior teeth [1]. While the mechanical properties, resistance to abrasion, and esthetic properties of composite resins have improved significantly during the last few years, their polymerization shrinkage still remains a challenge. Marginal discrepancies and microleakage [2], marginal discoloration, postoperative sensitivity, and secondary caries [3] are the consequences of polymerization shrinkage which ultimately limits composite resins application in direct restorations [4].

Recently, Ivoclar Vivadent has introduced a tooth-colored filling material, named Cention N, for the bulk application in retentive preparations with or without the application of an adhesive system [5]. Cention N is an “alkasite” restorative material reflecting a new category of filling material as a subgroup of the composite resins [6]. Cention N is a UDMA-based, self-cure material with optional additional light-curing which consists of a powder and a liquid component [7]. The liquid is composed of dimethacrylates and initiators and the powder consists of glass fillers, initiators, and pigments. Cention N entails a high-density polymer network and degree of polymerization over the complete depth of the restoration because of its cross-linking methacrylate monomers combined with a stable self-cure initiator [6].

The results of a previous study on Cention N's ability to prevent demineralization of enamel and dentin showed that this material prevents the recurrence of caries in the margins of restoration in a clinical setting [8]. The long-term release of fluoride and calcium ions from Cention N in acidic conditions has been reported to be the highest in comparison with conventional GIC [9]. Moreover, in a study conducted by Soumita *et al.* on the microleakage of class V cavities filled with flowable resin composites, glass ionomer cement, and Cention N, lowest amount of microleakage was reported for Cention N [6]. Similarly, Meshram et al., in their study assessing microleakage around class V cavities, showed that Cention N with adhesive had lower microleakage compared to the tested flowable composite. A previous study on the comparison of proximal contact tightness between two different restorative materials also showed that Cention N used as restorative material had a proximal contact tightness similar to that of the tested composite material [10]. Furthermore, previous investigations have declared superior microhardness and fracture resistance for Cention N when compared to amalgam [11, 12]. With regard to compressive strength, a recent study by Kumar and Ajitha [13] found no statistically significant difference between Cention N and amalgam.

To the best of the authors' knowledge, no study has previously investigated the effect of cavity size on fracture strength and marginal adaptation Cention N restorations with or without bonding. Therefore, in this study, we aimed to compare the fracture strength and marginal adaptation of MOD cavities restored with Cention N with or without bonding and resin composite and also to investigate the effect of cavity size on these properties. The null hypothesis to be tested is that different cavity preparation sizes and the type of restorative material have no effect on the fracture strength and marginal adaptation of MOD restorations.

## 2. Materials and Methods

The study protocol was approved by the university's ethics committee (IR.SUMS.DENTAL.REC.1398.092). A total of 120 maxillary premolars extracted for orthodontic reasons were collected for this study. Written informed consent was obtained from the parents or guardians at the time of tooth extraction. The parents were informed about the purpose of the study, privacy preservation, and data anonymity. After visual inspection with ×20 magnification, teeth with any sign of decay, defect, or discoloration were excluded from the study. The teeth were first disinfected in 1% thymol for one week at 4°C and then stored in saline until use for up to 3 months after extraction.

The maximum buccopalatal dimension of each tooth was measured using a digital caliper (Absolute Caliper, Mitutoyo Kawasaki, Japan) prior to the intervention. The approximate buccopalatal width of the selected teeth was 9.5 ± 0.5 mm. All teeth were mounted separately in acrylic resin up to 2 mm below cementoenamel junction (CEJ).

The specimens were randomly divided into two groups (*n* = 60). Standardized MOD cavities were prepared in all teeth by a single trained operator and periodontal probe was used to measure cavity dimensions in every step of the preparation. The occlusal depth was 3 mm, the mesiodistal length at the bottom of the proximal box was 1.5 mm, and the gingival wall was located 1 mm below the CEJ. The entire buccal and lingual walls of the preparation were parallel to the long axis of the tooth. The pulpal and gingival walls were perpendicular to the long axis. In half of the specimens, the cavity was prepared conservatively with 2 mm width in the buccolingual direction. In the other half, the buccolingual width was extended to 4 mm. The cavosurface margins were prepared at 90 degrees, and all internal line angles were rounded. The facial and lingual walls of the cavity were also prepared parallel to each other. The cavities were prepared using a diamond bur (Jota Co., Ruthi, Switzerland) mounted on a high-speed hand piece under cooling water.

The teeth were then divided into three subgroups according to the type of restoration (*n* = 20): composite resin, Cention N, and bonded Cention N.

In the composite resin and bonded Cention N groups, teeth were first conditioned with 37% phosphoric acid (Denfil, Vericom, Korea) for 15 seconds and then rinsed for extra 15 seconds. Spare water was removed by means of a cotton pellet. Then, a two-step etch-and-rinse adhesive system (Adper Single Bond 2, 3M ESPE, USA) was implemented in two coats, using a microbrush, and the solvent was vaporized via 5-second gentle air flow. The specimens were then light-cured for 20 seconds by means of an LED curing unit with a wavelength range of 440–480 mm and emitting light intensity of 1500 mW/cm^2^ (Radii Plus LED; SDI, Victoria, Australia). Afterwards, Tofflemire matrix holder with metal band was placed around the teeth and the teeth were filled with either Z250 microhybrid composite (3M ESPE, Germany) or Cention N (Ivoclar Vivadent, Germany) in accordance with the manufacturer's instructions. For the Cention N group, the cavities were filled with Cention N without any prior bonding application. A putty index was taken from the occlusal surface before cavity preparation and it was used for contouring the occlusal layer of the restoration. The proximal margins of the restorations were polished using flexible disks (Sof-Lex Pop-on, 3M ESPE; St. Paul, MN, USA). A single operator carried out the entire bonding process in an environment with controlled temperature and humidity.

Subsequently, the teeth were thermocycled for 1000 cycles at 5 ± 2ºC/55 ± 2°C, with a 30-second dwell time and a 5-second transfer time.

### 2.1. Fracture Strength

Half of the specimens in each subgroup (*n* = 10) were subjected to an axial compressive force using a universal testing machine (Zwick/Roell Z020, Germany). The force was applied by a steel ball with diameter of 8 mm at a strain rate of 0.5 mm/min parallel to the longitudinal axis of the tooth and contacting only on buccal and lingual cusps slope until the sample fractured. The force required to fracture the specimen was recorded in Newton. The fracture patterns were divided into two groups: restorable fractures in which the fractures stopped higher than 1 mm below the CEJ and unrestorable fractures in which the fractures stopped lower than 1 mm below the CEJ [14].

### 2.2. Marginal Adaptation

**Figure 1:**
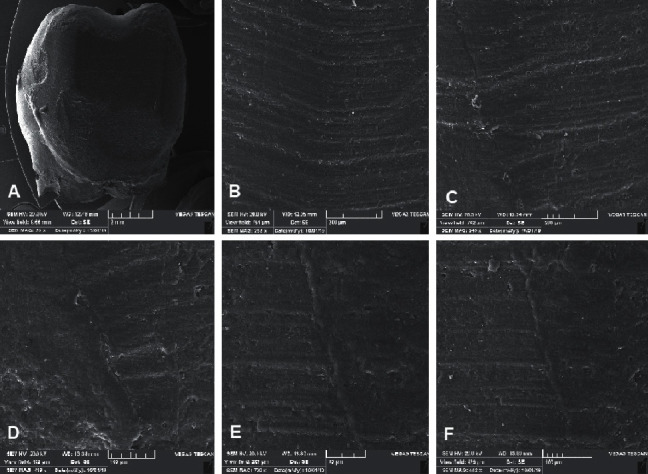
SEM images of the cavity-restoration interface at each gingival margin of the extensive restoration. (a) 20x, (b) 253x, (c) 249x, (d) 419x, (e) 709x, and (f) 442x.

**Figure 2:**
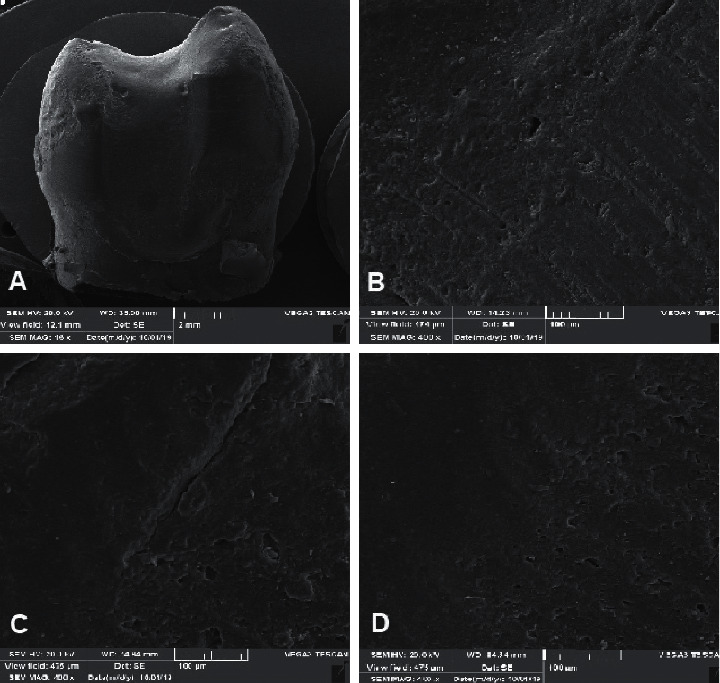
SEM images of the cavity-restoration interface at each gingival margin of the conservative restoration. (a) 16x, (b) 400x, (c) 400x, and (d) 400x.

Impressions were taken from the mesial and distal surfaces of the other half of the specimens (*n* = 10) using low-viscosity vinyl polysiloxane material (Express, 3M ESPE) [15]. These impressions were used to prepare replica in epoxy resin (EpoFix, Struers; Rodovre, Denmark). The replicas were coated with platinum for examination under scanning electron microscope. For quantitative margin evaluation, the interface of the cavity restoration was observed under ×400 magnification. At each gingival margin of the restoration, the cavity-restoration interface was divided into 5 sites for extensive restorations (Figure 1) and 4 areas for conservative restorations (Figure 2). Evaluation was performed by a technician in blinded condition. Marginal integrity was measured as a percentage of total margin length using the Adobe Photoshop CC 2016 software (Adobe Systems; Mountain View, CA, USA).

### 2.3. Statistical Analysis

The SPSS software version 18 (SPSS Inc., Chicago, IL, USA) was used to analyze data. Two-way ANOVA was applied to find interaction between the size and type of restoration. One-way ANOVA and post hoc Tukey's tests were used to detect significant differences in subgroup comparisons. Moreover, independent *t*-test was applied to observe the differences in fracture resistance and marginal adaptation values between two restoration sizes. The level of significance was considered as *P* ≤ 0.05.

## 3. Results

### 3.1. Fracture Resistance

Two-way ANOVA revealed a significant interaction effect between the size and type of restoration in terms of fracture resistance (*P* ≤ 0.001). Therefore, subgroup analysis using post hoc Tukey's test and independent t-tests was performed to investigate fracture resistance values between groups. Mean and standard deviations of fracture resistance values (N) in different experimental groups are presented in Table 1.

One-way ANOVA revealed significant difference in fracture resistance among the different types of conservative restorations (*P* ≤ 0.001). However, there was no significant difference among the extended restorations (*P* = 0.580). In the conservative restorations, bonded Cention showed the highest fracture resistance (1210.50 ± 230.97) which was significantly greater than Cention (882.70 ± 163.44) (*P* ≤ 0.001). However, there was no significant difference between fracture resistance of conservative composite (1052.30 ± 147.60) and that of bonded Cention or Cention (*P* = 0.150 and *P* = 0.120, respectively).

To analyze the effect of preparation size, independent *t*-test was conducted. The results showed that the conservative preparations had significantly greater fracture resistance compared to extended restorations in bonded Cention and composite groups (*P* ≤ 0.001). However, there was no significant difference between fracture resistances of conservative and extended Cention restorations (*P* = 0.300).

### 3.2. Marginal Adaptation

Two-way ANOVA revealed a significant interaction effect between the size and type of restoration in terms of marginal adaptation (*P* < 0.05). Among the conservative preparations, there was no significant difference between marginal adaptations of different types of restorations (*P* = 0.230). In the extended restorations, a significant difference was found between different types of restorations in terms of marginal adaptation (*P* ≤ 0.001). Therefore, subgroup analysis using post hoc Tukey's test was performed. Means and standard deviations of marginal adaptation are presented in Table 2.

In the extended group, composite showed the lowest marginal adaptation (81.45 ± 7.54) which was significantly lower than Cention (95.15 ± 5.40) and bonded Cention (90.95 ± 7.28) (*P* ≤ 0.001 and *P* = 0.040, respectively). There was no significant difference between Cention and bonded Cention in terms of marginal adaptation (*P* = 0.490).

Independent *t*-test showed that conservative composite restorations have significantly greater marginal adaptation compared to extended composite restorations (*P* ≤ 0.001). However, there was no significant difference between marginal adaptations of conservative and extended restorations in the Cention or bonded Cention groups (*P* = 0.440 and *P* = 0.120, respectively).

## 4. Discussion

The present study investigated the fracture strength and marginal adaptation of conservative and extended conventional Cention N, bonded Cention N, and resin composite restorations. The study hypotheses were partially rejected, because the type of the restorative material and the volume of cavity preparation influenced fracture strength and marginal integrity in some situations.

Microleakage still remains a quite major concern in clinical restorative dentistry. Two of the most common reasons of restoration failure, sensitivity and secondary caries, can occur as the result of microleakage [16]. While various *in vitro* methods have been introduced to evaluate the marginal adaptation, currently, no specific method is determined as superior to other techniques in terms of measuring microleakage and predicting the clinical performance of restorations margins [17, 18]. While the dye penetration method is simple and inexpensive, it is devoid of clinical relevance and interstudy comparability [19]. On the other hand, the replica SEM technique is a well-documented method that offers both qualitative and quantitative assessments of margin integrity [20]. Therefore, it was implemented in this study to investigate the marginal adaptation of the restorative materials. Epoxy resin was used in the current study as it adequately replicates the details of silicone impressions of dentin surfaces in the *in vitro* setting [18, 21]. However, there are shortcomings to the replica technique including the accuracy of the impression, as well as a weak-to-moderate correlation to clinical findings [18].

The results of the present study indicated no difference between marginal adaptations of different restorative choices when the cavity volume was conservative. However, our findings demonstrated that when the cavity preparation size extends, the type of material plays a significant role with regard to the amount of marginal adaptation. Superior marginal adaptation for Cention N, whether conventional or bonded, over composite resin was recorded in this study. This finding is also in agreement with a previous study by Soumita et al. on the microleakage of class V cavities, which showed lower amount of microleakage for Cention N compared to the tested resin composite [6]. According to the manufacturer, the resin composite tested in this study manifests a polymerization shrinkage more than 2% [22]. Hence, lower marginal adaptation for composite resin was expected due to the marginal and internal microleakage as the result of polymerization shrinkage stress. Moreover, Mazumdar et al. [11] proposed Cention N as a new restorative material with lower microleakage compared to amalgam and glass ionomer cement. The fillers of Cention N include ytterbium trifluoride, barium aluminum silicate glass filler, a calcium barium aluminum fluorosilicate glass filler, an isofiller (Tetric N-Ceram technology), and a calcium fluorosilicate (alkaline) glass filler [23]. It seems that the low amount of microleakage in Cention N restorations is due to its specially patented isofiller which is partially functionalized by silanes and leads to a minimum shrinkage stress. This isofiller keeps the shrinkage force at a minimized level since it acts as a shrinkage stress reliever [24]. Increased occurrence of nonrestorable fractures was observed in the extended composite group which can be attributed to the accumulation of polymerization shrinkage stress in the remaining week's dental structure. Most of the fractures in the bonded Cention N group were restorable.

Among the conservative groups, the bonded Cention group showed higher fracture strength compared to the Cention group. It seems that using bonding agent can strengthen the remaining tooth structure. This finding was in accordance with a previous research which showed improved fracture resistance in bonded amalgam restorations compared to the conventional amalgam [25]. The fracture strength of Cention N, whether conventional or bonded, was comparable to that of the tested resin composite in both conservative and extended restorations. In line with our findings, another study which investigated the fracture resistance of three different restorative materials, Z350 nanofill composite resin, Cention N, and silver amalgam material in a class II cavity, concluded that the use of Cention N and Z350 restorative materials significantly strengthens teeth after class II cavity preparation and restoration [12]. Moreover, Sharma et al. [26] showed similar fracture resistance readings for Cention N and Z350 composite in endodontically treated teeth. The high filler contents of barium aluminum silicate glass and calcium aluminum silicate glass can be a potential reason for this high and comparable strength of Cention N [26].

Reduced cavity size led to the improvement of fracture strength for bonded Cention N and composite. This was expected as the remaining sound tooth structure plays a vital role in the fracture strength of the teeth [27]. This finding is of high clinical importance and should be taken into account when preparing a MOD cavity. Lee et al. [28] related cuspal deflection to the width and depth of the cavity but Forster et al. [29] showed that when the cavity depth was 3 mm, increasing the width of the cavity did not alter the fracture strength. They concluded that, in shallow preparations, the thickness of the walls is not important. The difference between the results of the two studies can be attributed to the different preparation geometry. In our study, the depth of the preparation in proximal boxes was much more than 3 mm. Deep gingival floors of the proximal boxes which could act as a fulcrum for cusp bending in conjunction with decreased thickness might lead to lower fracture strength in extended preparations [30]. Another study by Pottemaier et al. [31] showed that increasing the cavity width from 1/3 of the intercuspal distance to 2/3 did not decrease the fracture strength.

Comparing conventional Cention N and bonded Cention N, our findings failed to show a significant improvement in marginal adaptation with the application of prior bonding. Regarding fracture strength only in the conservative groups, the fracture strength of the bonded Cention N was significantly higher than that of the conventional Cention N. In a study conducted by Meshram et al. [24], lower microleakage was seen with Cention N with adhesive compared to Cention N without adhesive. A possible explanation for the discrepancy observed between our finding and that of Meshram et al. could be due to a different method of microleakage measurement. While the replica SEM technique was used in the present study to measure marginal adaptation, Meshram et al. adopted the dye penetration method. Since bonded Cention N restorations are costlier and laborious, their routine application in dental practice cannot be recommended until further advantages are verified by future studies.

Our results are obtained with some limitations. Cyclic mechanical loads generated during chewing are different from the static compressive force applied during the fracture strength test. Other limitation of the current study is rigid fixation of the sample teeth instead of simulating periodontal ligaments and tooth supporting tissues. This can influence the results of mechanical tests. Hence, future research is required to evaluate the performance and other mechanical properties of this alkasite restorative material in more clinically relevant settings.

## 5. Conclusion

The volume of the cavity preparation was shown to be effective in the materials fracture strength and marginal adaptation. Cention N showed promising results in terms of fracture strength and marginal adaptation in either conventional or extended MOD cavities.

## Figures and Tables

**Table 1:** Mean ± SD of fracture resistance (N) in the experimental groups and fracture pattern (restorable/nonrestorable).

Type of restoration	Size of restoration		*P* value
	Conservative	Extended	
Cention	882.70 ± 163.44^b, A^ (5/5)	804.90 ± 165.90^a, A^ (4/6)	0.300
Bonded Cention	1210.50 ± 230.97^a, A^ (7/3)	760.50 ± 85.66^a, B^ (7/3)	≤0.001
Composite	1052.30 ± 147.60^ab, A^ (5/5)	816.30 ± 108.95^a, B^ (2/8)	≤0.001
*P* value	≤0.001	0.580	—

Different lower case letters show significant difference in each preparation size (in a column). Different upper case letters show significant difference in each type of restoration (in a row).

**Table 2:** Mean ± SD of marginal adaptation (%) of experimental groups.

Type of restoration	Size of restoration		*P* value
	Conservative	Extended	
Cention	93.01 ± 4.54^a, A^	95.15 ± 5.40^a, A^	0.440
Bonded Cention	96.38 ± 4.60^a, A^	90.95 ± 7.28^a, A^	0.120
Composite	92.52 ± 4.06^a, A^	81.45 ± 7.54^b, B^	≤0.001
*P* value	0.230	≤0.001	—

Different lower case letters show significant difference in each preparation size (in a column). Different upper case letters show significant difference in each type of restoration (in a row).

## Data Availability

The data used to support the findings of this study are included within the article.
